# Fibroblast growth factor receptor risk signature predicts patient prognosis and immunotherapy resistance in colorectal cancer

**DOI:** 10.3389/fimmu.2024.1493673

**Published:** 2024-11-29

**Authors:** Xiaofang Li, Zhiling Pan, Tiankuo Luan, Qian Xiao, Liuying Li, Qianxue Wu, Guoqing Yao, Xiang Zhang, Daqiang Song

**Affiliations:** ^1^ Department of Pharmacy, The Affiliated Hospital of Southwest Medical University, Luzhou, Sichuan, China; ^2^ Department of Operating Room, Affiliated Hospital of Youjiang Medical University for Nationalities, Baise, China; ^3^ Baise Key Laboratory of Molecular Pathology in Tumors, Baise, China; ^4^ Chongqing Key Laboratory of Molecular Oncology and Epigenetics, The First Affiliated Hospital of Chongqing Medical University, Chongqing, China; ^5^ Department of Vascular Surgery, The First Affiliated Hospital of Chongqing Medical University, Chongqing, China; ^6^ Department of Breast and Thyroid Surgery, The First Affiliated Hospital of Chongqing Medical University, Chongqing, China

**Keywords:** fibroblast, tumor immunity, prognosis, therapy resistance, colorectal cancer

## Abstract

**Background:**

Fibroblast Growth Factor Receptor (FGFR) signaling is linked with tumor progression and tumor immunoevasion, yet the potential effect of FGFR signature on the prognosis of patient with colorectal cancer (CRC) and response to immune therapy remains elusive.

**Methods:**

The fibroblast growth factor receptor risk signature (FRS) was identified through single-cell RNA sequencing, bulk RNA sequencing, and machine learning techniques. Signaling enrichment analyses were conducted using Gene Set Enrichment Analysis (GSEA) and the Kyoto Encyclopedia of Genes and Genomes (KEGG). Drugs targeting the FRS were predicted using the Cancer Therapeutics Response Portal (CTRP) and PRISM databases. The analysis of T cell function and the tumor microenvironment (TME) was performed using flow cytometry.

**Results:**

In this study, we characterized the FRS in cancer patients with CRC. By integrating advanced techniques, we identified the FRS and revealed the intricate molecular landscape and diversity of the FRS within the TME. Notably, the FRS effectively predicted unfavorable prognosis and resistance to immunotherapy in CRC patients. Furthermore, PHA-793887, identified as a potential FRS inhibitor by the CTRP and PRISM databases, significantly restructured the immunosuppressive TME and enhanced the antitumor immune response, resulting in a reduced tumor burden in the MC38 murine tumor model.

**Conclusion:**

Together, these data support FRS positively correlates with poor prognosis and therapy resistance. The PHA-793887 could be a potential FRS inhibitor to improving the effectiveness of CRC management via bolstering antitumor immunity.

## Introduction

Colorectal cancer (CRC) stands as a significant contributor to cancer-related deaths globally, featuring an intricate interaction between genetic and environmental elements that propel its onset and advancement (1, 2). The disease’s diversity and inconsistent reaction to therapies highlight the necessity for a more profound comprehension of its molecular foundations. Despite advancements in early detection and treatment, the precise mechanisms driving CRC progression and the genes influencing patient prognosis remain insufficiently understood. Elucidating these complexities is essential for developing new prognostic indicators and treatment strategies.

Fibroblast growth factor receptor (FGFR) signaling plays a critical role in multiple aspects of tumor biology, including cell proliferation, survival, angiogenesis, and invasion. The activation of FGFR signaling is observed in various cancers, contributing to aggressive disease phenotypes and poor outcomes (3–5). Nevertheless, the correlation between FGFR signaling and the tumor microenvironment (TME) in CRC remains inadequately defined. With an increasing understanding of the TME’s influence on cancer progression and response to immunotherapy, clarifying the association between FGFR signaling and the immunological landscape of CRC is highly relevant (6, 7). Examining FGFR risk signature (FRS) holds promise for shedding light on CRC pathogenesis and guiding prognostic assessments of patients.

The emergence of cancer immunotherapy has revolutionized the management of various malignancies, including CRC. Despite its potential, the clinical effectiveness of immunotherapy is frequently impeded by the development of treatment resistance, benefiting only a subset of patients (8, 9). The mechanisms driving this resistance are complex, involving aberrant activation of specific intracellular transcriptional pathways in tumor cells, among other factors (10, 11). In the context of CRC, our investigation has revealed a significant association between the FRS and immunotherapy resistance. Given the established role of FRS in influencing cellular behavior and the TME, targeting this signature presents a promising strategy to overcome this resistance.

In this study, we utilized bioinformatic tools and experimental models to identify and forecast the effectiveness of inhibitors targeting the FRS. Our goal was to devise a therapeutic strategy capable of selectively disrupting FRS, thereby enhancing the anti-tumor immune response and impeding tumor progression. Our results indicate that FRS-targeting inhibitors have the potential to reshape the TME, enhance T cell function, and elicit antitumor responses. This discovery carries substantial translational significance, suggesting that the integration of FRS inhibitors with existing immunotherapies may provide a novel approach to improving treatment efficacy and clinical outcomes for CRC patients.

## Materials and methods

### Animal experiments

In the present investigation, male C57BL/6J mice, aged 6-8 weeks, were obtained from Ensiwer Corporation and utilized in the ICB-resistant MC38 tumor model. The colorectal cancer MC38 cell line, adjusted to a concentration of 1 × 10^5 cells, was subcutaneously inoculated into these mice. On the seventh day post-inoculation, the animals were administered either IgG or anti-CTLA4 antibodies (Bio X cell, 9H10) at a dosage of 5 mg/kg for a continuous period of five days. At the culmination of the designated experimental timelines, the tumor tissues were meticulously excised for further analysis. In a subsequent experimental paradigm, mice bearing MC38 tumors were subjected to treatment with either a control vehicle or PHA-793887 (MedChemExpress, HY-11001), administered at a dosage of 10 mg/kg. Post-treatment, the excised tumor tissues underwent flow cytometric analysis to elucidate the impact of PHA-793887 on the TME. Concurrently, tumor dimensions were meticulously monitored biweekly throughout the experimental period.

### Flow cytometry analysis

Live cells were assessed using the Fixable Viability Dye eFluor 450. To evaluate cytokine production, the cells were stimulated with the Cell Stimulation Cocktail and subsequently labeled with anti-IFN-γ (BioLegend, XMG1.2) and anti-TNF-α (BioLegend, MP6-XT22) antibodies. Other antibodies included an-CD45 (Biolegend, 2D1), anti-CD11b (BioLegend, M1/70), anti-CD8a (BioLegend, 53-6.7), anti-CD4 (BioLegend, GK1.5), anti-FOXP3 (BioLegend, MF-14), anti-F4/80 (BioLegend, BM8).

Analysis of stained cells was conducted using a BD FACSCanto II Flow Cytometer in conjunction with BD FACSDiva software (BD Biosciences), and the resulting data were processed using FlowJo software (version 10.5.3).

### RNA sequencing analysis

Total RNA was isolated from MC38 tumor tissues using the Trizol reagent (Invitrogen, catalog number 15596026). RNA samples were forwarded to ANNOROAD for construction of sequencing libraries and subsequent sequencing on the NovaSeq platform (Illumina). The resultant raw fastq files were processed to quantify gene expression as transcripts per million (TPM) using the htseq-count tool, facilitating the downstream analysis. Differential expression analysis of genes (DEGs) was performed using the “DESeq2” R package, applying stringent filtering criteria: a fold change threshold greater than 2, an adjusted *P*-value of less than 0.05, and a mean log2-TPM in the high-expression cohort exceeding 0.

### Data acquisition

In the present investigation, we procured five distinct public datasets from the NCBI Gene Expression Omnibus (GEO) repository. Our approach entailed the application of scRNA-seq datasets, namely GSE231559 and GSE166555, to dissect the heterogeneity of fibroblast populations within both normal and neoplastic colorectal tissues. Additionally, we leveraged the COAD cohort from the TCGA database and an aggregate of three bulk RNA sequencing datasets, GSE17536, GSE29621, and GSE38832, for the development and substantiation of our prognostic model. It is important to note that for analyses based on publicly available datasets, neither patient consent nor ethical board approval is required.

### scRNA-seq data analysis

The processing of scRNA-seq data was executed utilizing the ‘Seurat’ R package (version 5.0.2), following established protocols (12). Initially, a stringent filtration criterion was applied, excluding cells with gene expression levels below the 200-gene threshold or exceeding the 6000-gene ceiling, as well as those with mitochondrial gene expression surpassing the 5% mark. This exclusionary process was pivotal for the retention of a substantial cell population representative of the datasets in question. Subsequently, the SCTransform function was utilized to standardize and normalize the raw count data, which was then subjected to principal component analysis (PCA) to identify the underlying patterns. To address and neutralize batch effects inherent in the dissociated scRNA-seq datasets, the “Harmony” R package, was strategically implemented. Clustering was conducted by assessing the edge weights connecting pairs of cells, culminating in the construction of a shared nearest-neighbor graph. This graph was adeptly derived using the Louvain algorithm, facilitated by the FindNeighbors and FindClusters functions. The outcome of this process was a visual representation of the cells, rendered through the UMAP algorithm, providing a comprehensive overview of cellular distribution. The “FindMarkers” function was employed to pinpoint genes that were preferentially expressed within specific clusters, in addition to identifying DEGs. Each resultant cell cluster was annotated with reference to established cell-type marker genes, enhancing the interpretability of the data. To elucidate the distinct expression profiles of the identified genes at the single-cell level, the “scRNAtoolVis” package was utilized, providing a graphical interface that enabled precise and clear visualization of gene expression patterns.

### High dimensional weighted gene co-expression network analysis

To explore genes associated with FGFRS-positive fibroblasts, we conducted a hdWGCNA using the “hdWGCNA” package. We created metacells for each sample and cell cluster, with 50 cells per metacell, and applied a standard pipeline of functions to analyze gene expression patterns and visualize module relationships in a reduced-dimensional space.

### Machine learning-based construction of an FGFRS-fibroblast-related prognosis model

As previous published study (13, 14), we identified FGFRS-fibroblast-related genes using hdWGCNA and validated their predictive potential in tumor development with three transcriptome datasets, GSE17536 GSE29621, and GSE38832. Using the “mlr3” package, we developed a predictive model incorporating seven machine learning algorithms. Following cross-validation, we selected the most accurate model and assessed its predictive ability on an independent test set.

### Trajectory and cell-cell communication analysis

Employing an unsupervised approach, pseudotemporal analysis was performed using the “Monocle” package, applying the DDR-Tree algorithm with its default parameters to delineate the developmental trajectory of fibroblasts. Following the pseudotemporal trajectory mapping, the “plot_pseudotime_heatmap” function was engaged to craft a heatmap. This visual tool effectively depicted the fluctuating expression patterns of a cohort of genes, illustrating their dynamic behavior along the pseudotime trajectories of fibroblasts. Furthermore, to uncover potential cellular interactions, both intracellular and extracellular, the “CellChat” package was deployed using its default settings and recommended pipeline configurations. This application facilitated the identification of communication networks among fibroblasts and other cellular components within the TME.

### Enrichment analysis

The Seurat package’s “FindMarkers” function was deployed to discern DEGs within each delineated cell subcluster. The selection criteria for these genes were stringent, requiring a fold change surpassing a threshold of 2 and an adjusted p-value below the significance level of 0.05. Subsequently, leveraging the identified DEGs, a comprehensive GSEA and KEGG enrichment analyses were conducted to explore the functional profiles of the cell subgroups. These analyses were executed utilizing the “clusterProfiler” package, which provided a robust framework for assessing the overrepresentation of specific gene sets and biological processes. To visually represent the functional enrichment results, the “GseaVis” package was employed. This tool facilitated the creation of intuitive and informative visualizations that encapsulated the enriched biological themes and pathways associated with the cell subclusters under investigation.

### Non–negative matrix factorization analysis

To explore the diversity of FGFRS subtypes, we applied the NMF algorithm from the “NMF” package (15). The objective was to identify distinct subtypes characterized by unique gene expression patterns. To assess the prognostic value of these genes, we conducted survival analysis using the “survival” package. Additionally, we used the “ggrisk” package to analyze the survival and risk profiles of cancer patients, categorizing them into high- and low-risk groups. The analyses were considered statistically significant for *P* values below 0.05.

### Statistical analysis

All computational analyses and graphical representations were executed utilizing the R software (v4.3.2). The strength and direction of the linear relationships between pairs of continuous variables were evaluated through Pearson’s correlation coefficients. In the case of quantitative datasets, statistical comparisons among subgroups were made using either a two-tailed, unpaired Student’s t-test for two-group comparisons or a one-way analysis of variance (ANOVA) complemented with Tukey’s *post hoc* test for multiple group analyses. The threshold for statistical significance was set at a *P*-value of less than 0.05.

## Results

### Single-cell RNA sequencing analysis reveals FGFRS-positive fibroblast subsets

**Figure 1:**
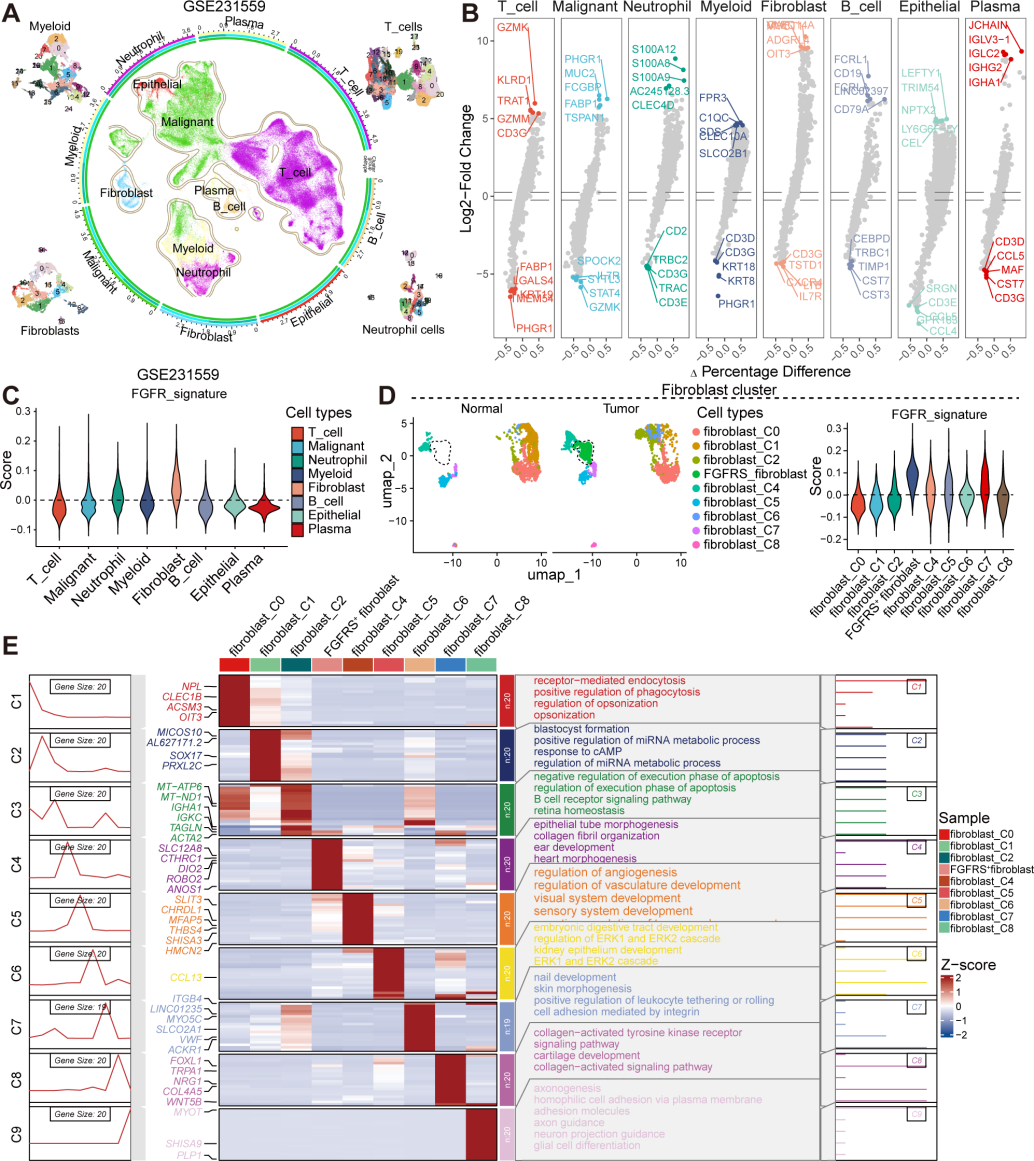
The scRNA-seq analysis uncovers the diversity of fibroblast subsets within CRC. **(A)** UMAP plot representing 8 distinct groups. The color coding corresponds to predominant cell types, with contours delineating cluster boundaries. The four corner insets provide a detailed view of myeloid, T cells, fibroblasts, and neutrophils. The peripheral axis displays the log-transformed total cell counts per class. The concentric colored tracks (exterior to interior) signify class identity (aligned with the central UMAP), cluster, group, and cell types. **(B)** Volcano plot showing the differential expression of markers in the distinct cell types. **(C)** Violin plot showing the score of FGFRS in the distinct cell types. **(D)** UMAP plot showing the fibroblast subsets in the normal or tumor tissues (left) and the score of FGFRS in the distinct fibroblast subsets (right). **(E)** The left panels illustrate the dynamic patterns of DEGs specific to each subset, while the central heatmap compares the expression profiles of these DEGs across populations. The right panels summarize the GO terms, providing insights into the biological functions associated with each cluster.

To elucidate the role of the fibroblast growth factor receptor (FGFR) signature in tumors, we analyzed single-cell RNA sequencing (scRNA-seq) data from colorectal cancer contained in the Gene Expression Omnibus (GEO) database (GSE231559, GSE166555) to identify potential fibroblast subsets that are positive for FGFRS (FGFRS^+^). In the GSE231559 dataset, we annotated cell types using established markers, including T cells, malignant cells, neutrophils, myeloid cells, fibroblasts, B cells, epithelial cells, and plasma cells, each characterized by their unique gene expression profiles (**Figure 1A**). Notably, the analysis highlights genes like GZMK, GZMM, CD3G, which are expressed in T cells, and genes such as CD79A and CD19, which are characteristic of B cells. The myeloid and plasma cells are associated with genes like C1QC and IGHA1, respectively (**Figure 1B**). In the GSE166555 dataset, we identified 22 clusters and further annotated these clusters to 10 main clusters based classic markers, including B cells, DC cells, endothelial cells, fibroblasts, malignant cells, mast cells, monocyte/macrophage, plasma and T cells (**Supplementary Figure S1A**). These established markers including of MZB1, MS4A1, EPCAM, CD3D, CPA3, CD163, COL1A2, VWF, and IDO1 (**Supplementary Figure S1C**). Significantly, each cluster exhibited different gene expression profiles (**Supplementary Figure S1D**). Furthermore, to analyze the effect of FGFR signaling, we constructed an FGFR signature (FGFRS) using 86 genes involved in the FGFR signaling pathway from Molecular Signatures Database (MSigDB). This signature was then applied to score the identified cell groups using the “AddModuleScore” function in the Seurat package. Our findings reveal that fibroblast cell groups exhibit the highest FGFRS score, indicating a significant involvement of FGFR signaling in these cells (**Figures 1C**, **Supplementary Figure S1B**). To further dissect the role of FGFRS in fibroblast cell cluster, we isolated these cells and identified nine main fibroblast subgroups. Notably, in tumor tissues, a distinct fibroblast subgroup was found to have an increased proportion relative to normal tissues and showed highest FGFRS score (here after as “FGFRS^+^ fibroblast”) (**Figures 1D**, **Supplementary Figure S1E**). This observation suggested a potential role of FGFRS in the transformation and proliferation of fibroblasts in the TME. Furthermore, Gene Set Enrichment Analysis (GSEA) was performed on the transcriptome of this specific fibroblast subsets. The enrichment analysis identified several key cells signaling pathways that are potentially modulated by FGFRS. In the GSE231559 dataset, these pathways include receptor-mediated endocytosis, positive regulation of phagocytosis, and the regulation of miRNA metabolic processes, among others. In the FGFRS^+^ fibroblast, some signaling pathways were significantly enriched, including epithelial tube morphogenesis and collagen fibril organization (**Figure 1E**). In the GSE16655 dataset, FGFRS^+^ fibroblasts exhibited upregulated signaling pathways, including cytokine-cytokine receptor interactions, while downregulated pathways included the Wnt and Hippo signaling pathways (**Supplementary Figure S1F**). In summary, the integration of scRNA-seq data analysis highlighted that FGFRS is enriched in the fibroblast cluster within the TME, and these relevant signaling pathways may be critical for the role of FGFRS^+^ fibroblasts in tumor progression.

### Predictive model to identify core genes correlated to FGFRS^+^ fibroblasts

**Figure 2:**
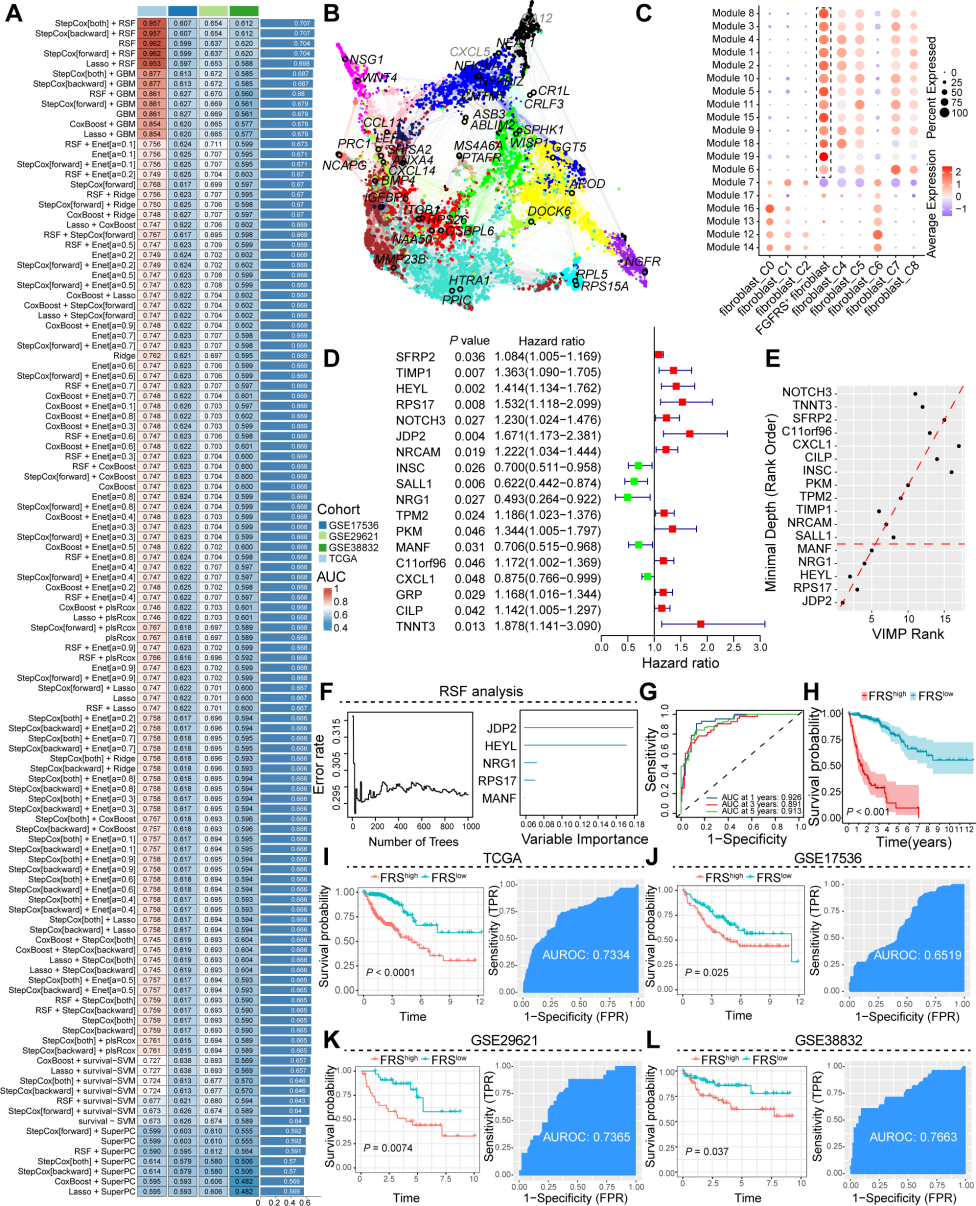
Construction and validation of the artificial intelligence-derived prognostic model. **(A)** Assessment of the area under the receiver operating characteristic curve (AUC) for multiple models generated from diverse combinations of machine learning algorithms across four cohorts. **(B)** Illustration of the interaction network among core prognostic genes, highlighting their interconnectivity. **(C)** Dot plot representation of the expression levels of module genes across distinct fibroblast subpopulations. **(D)** Forest plot delineating the hazard ratios associated with prognostic genes, quantifying their impact on patient outcomes. **(E, F)** RSF analysis, emphasizing the relative importance of prognostic genes in predicting survival. **(G)** The AUC curve at various time points, demonstrating the predictive accuracy of the FGFRS for survival. **(H)** Kaplan-Meier survival curves comparing the overall survival of cancer patients stratified by high and low FGFRS scores, FRS, FGFR risk score. **(I-L)** A comprehensive analysis was conducted to evaluate the survival benefits and predictive efficacy of the FSR score in cancer patients using data from TCGA and the GEO. The analysis was stratified according to high and low FRS expression.

To identify potential core genes associated with FGFRS^+^ fibroblasts, we conducted high-dimensional Weighted Gene Co-expression Network Analysis (hdWGCNA), a comprehensive methodology for analyzing co-expression networks in the scRNA-seq data. This analysis aimed to detect co-expressed gene modules and unravel their functional roles within FGFRS^+^ fibroblasts. Subsequently, we constructed a scale-free co-expression network by applying an optimal soft thresholding power of 12 (**Supplementary Figure S2A**). From this analysis, we distinguished a total of 19 distinct gene co-expression modules, identified the top 10 hub genes from these modules, and constructed protein-protein interaction (PPI) networks for the identified hub genes in each module (**Supplementary Figure S2B, C**, **Figure 2B**). Additionally, we investigated the correlation between each module (**Supplementary Figure S2D**), where modules 1, 2, 3, 4, 5, 6, 8, 9, 10, 11, 15, 18, and 19 displayed significant activation primarily in FGFRS^+^ fibroblasts (**Figure 2C**, **Supplementary Figure S2E**). Subsequently, 325 genes from these modules underwent univariate Cox regression analysis in the TCGA COAD cohort, leading to the identification of 13 genes significantly associated with overall survival in CRC patients (**Figure 2D**). Additionally, by integrating data from the TCGA COAD and GEO database cohorts, we constructed robust models using 101 algorithmic combinations and calculated the area under the curve (AUC) for each model across all cohorts to assess their predictive capacity. (**Figure 2A**). Among the 101 models, the Step Cox (direction = both) algorithm in conjunction with a Random Survival Forest (RSF) demonstrated the highest AUC, serving as the basis for the final model creation. We further utilized RSF analysis to assess the prognostic relevance of various genes in predicting patient survival outcomes systematically. Notably, genes such as JDP2, HEYL, NRG1, RPS17, and MANF exhibited substantial predictive value, as indicated by their low Minimal Depth and high Variable Importance scores, thus influencing the accuracy of the survival model significantly (**Figures 2E, F**, **Supplementary Figure S2F**). The gene signature, comprising JDP2, HEYL, NRG1, RPS17, and MANF, known as the FRS, has demonstrated substantial predictive capabilities for patient survival at the 1-year, 3-year, and 5-year intervals. This integrated genetic profile serves as a valuable prognostic tool, providing insights into anticipated clinical outcomes for patients over the specified time frames (**Figure 2G**). Based on the FRS, cancer patients were categorized into high- and low-risk groups, with high-FRS patients exhibiting a poor prognosis (**Figure 2H**). These results were further validated using cohorts from the TCGA and GEO databases. In the TCGA cohort, Kaplan-Meier survival curve analysis revealed adverse outcomes for high-FRS patients, with an Area Under the Receiver Operating Characteristic curve (AUROC) of 0.7334 serving as a critical metric for model evaluation. These comprehensive results highlight the predictive capacity of the model and align with the analysis of COAD cohorts from the GEO database (**Figures 2I-L**).

### FRS is correlated to the tumor immunity

**Figure 3:**
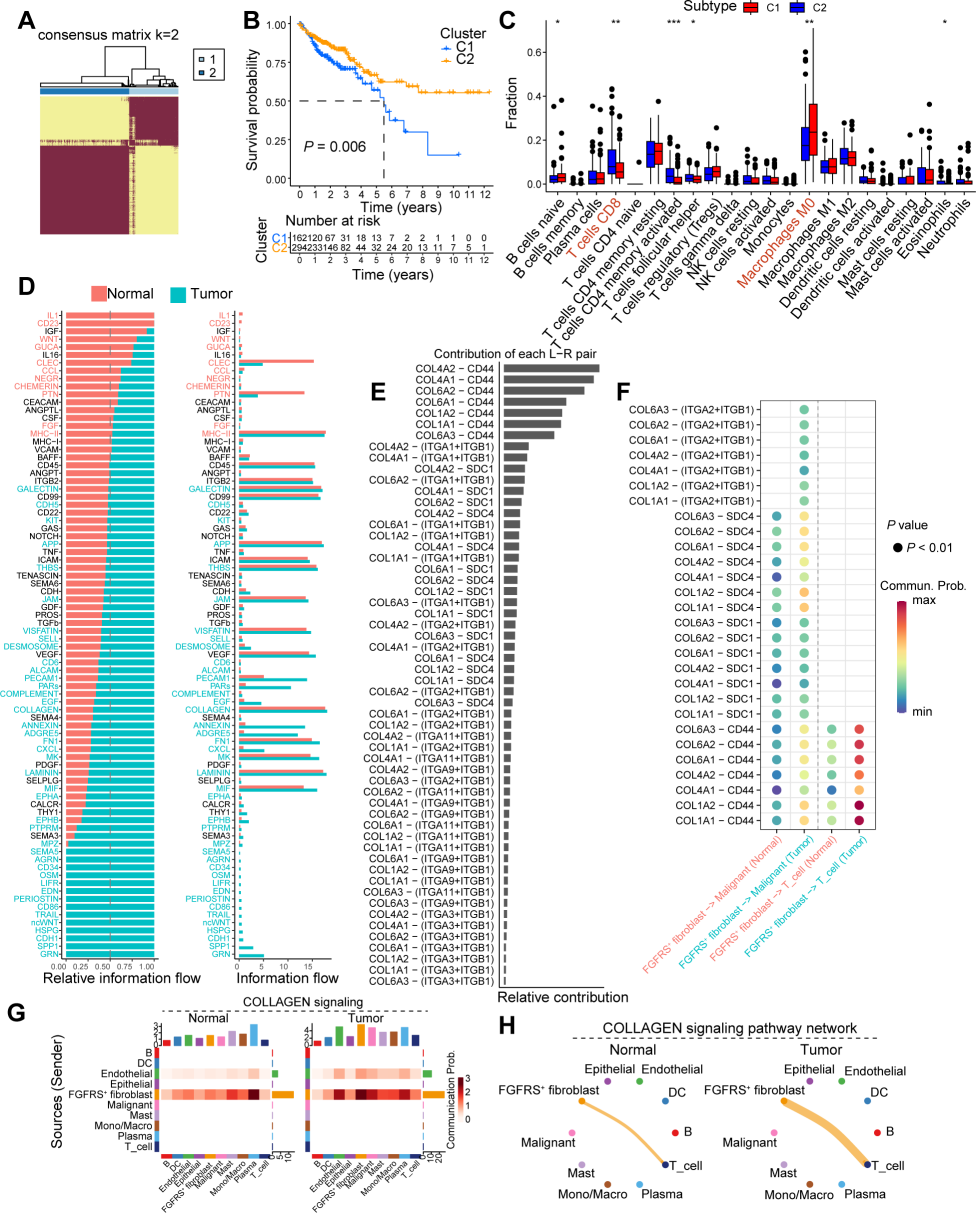
FGFRS-related molecule subtype correlates to tumor immunity. **(A)** Heatmap showing the clustering outcomes derived from NMF analysis, which has been applied to categorize patients into two distinct molecular subtypes based on the FGFRS signature. **(B)** Survival analysis of cancer patients in the cluster 1 and cluster 2. **(C)** Box plot showing the fractions of immune cells in the COAD tissues between cluster 1 and cluster 2. **(D)** The relative flow among cell clusters in the TME of normal and COAD tumors. **(E)** The contribution of ligand-receptor pair in the TME of COAD tissues. **(F)** The dot plot showing the interaction of ligand-receptor between FGFRS-positive fibroblasts and T cells. **(G)** Heatmap showing the changes of collagen signaling among cell clusters between normal and tumor tissues. **(H)** A shell plot illustrating the interaction of collagen signaling between FGFR score (FGFRS)-positive fibroblasts and T cells is presented. *P* values are from log-rank test **(B)** and two-way ANNOVA **(C)**. ^*^ *P* < 0.05, ^*^ *P* < 0.01, ^***^ *P* < 0.0001.

To further analyze the correlation between the FRS and tumor immunity, we employed Non-negative Matrix Factorization (NMF), a robust technique for clustering analysis, to investigate the FRS of cancer patients based on the FGFRS. Our analysis specifically aimed to identify the optimal number of clusters (K) that would best capture the heterogeneity within the patient cohort. Following the NMF-based clustering, we observed that when K was set to 2, the consensus matrix revealed a clear distinction between two distinct patient groups. This finding suggests that dividing the patients into two groups at K=2 provides the most meaningful separation in terms of their FRS (**Figure 3A**, **Supplementary Figure S3A**). Notably, patients in cluster 1 exhibited a poorer prognosis compared to those in cluster 2 (**Figure 3B**). Upon TME score analysis, cluster 1 displayed lower immune scores, including stromal score, immune score, and ESTIMATE score, in contrast to cluster 2 (**Supplementary Figure S3B**). Additionally, CIBERSORT analysis was conducted for both clusters. Notably, tumors from patients in cluster 1 demonstrated reduced CD8^+^ T cell infiltration but increased M0-like macrophages compared to those in cluster 2 (**Figure 3C**). Moreover, tumors from patients in cluster 1 exhibited decreased expression levels of immunostimulatory molecules (**Supplementary Figure S3C**). These findings imply that tumors from patients in cluster 2 might possess heightened antitumoral immunity within an immune-stimulating TME. The cell-cell interactions within the TME play a crucial role in tumor progression. Therefore, to further understand the interactions between FGFRS^+^ fibroblasts and T cells, we conducted CellChat analysis. While the total interaction strength was notably increased in tumor tissues compared to normal tissues, the number and weight of interactions showed no significant differences (**Supplementary Figures S3D, E**). Subsequently, we investigated whether specific signaling interactions between FGFRS^+^ fibroblasts and T cells were altered. Utilizing signal flow analysis, a quantitative method for assessing information transmission in biological systems, revealed substantial changes in signaling pathways between tumor and normal tissues. These results highlight a significant divergence in the flow of information within distinct tissue states (**Figure 3D**). In our subsequent analysis, we delved into the ligand-receptor interactions within the TME, focusing on the FGFRS^+^ fibroblasts and their potential binding partners. Utilizing a comprehensive approach, we identified a significant interaction between collagen (COL)-related ligands and the CD44 receptor on T cells. Notably, this interaction was found to be markedly enhanced in the TME compared to normal tissues (**Figure 3E**). Our findings underscore the complex interplay between the extracellular matrix components and immune cells. Specifically, the pairs COL4A2-CD44, COL6A1-CD44, and COL1A2-CD44 demonstrated a pronounced increase in their relative contribution to the signal transduction within the tumor context (**Figures 3F**, **Supplementary Figure S3F**). Moreover, there was a significant increase in COLLAGEN signaling between FGFRS-positive fibroblasts and T cells in the tumor tissue compared to normal tissues (**Figures 3G, H**). This heightened interaction between collagen ligands and the CD44 receptor on T cells implies a potential role in influencing the immune response within the TME.

### FRS were negatively correlated with T cell function

**Figure 4:**
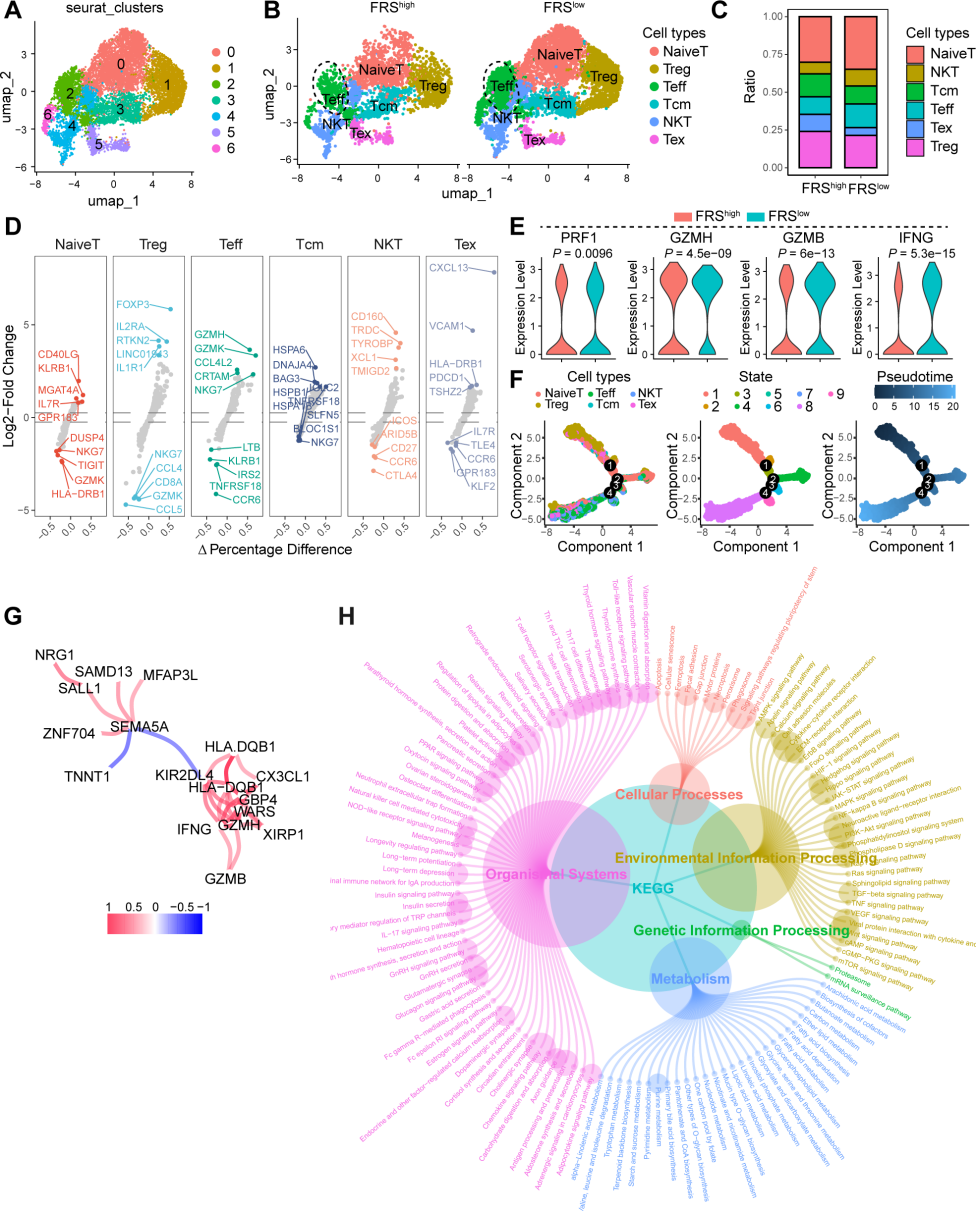
FRS correlates to the T cell function in the TME. **(A)** The UMAP depicting the cell cluster of T cells within COAD tumors, with each distinct color indicating a different cell type. **(B)** The UMAP visualizing the distribution of cell clusters in both FRS high and FAMS low COAD tumors, Treg, regulator T cells, Teff, effector T cells, Tcm, central memory T cells, NKT, natural killer cells, Tex, exhausted T cells. **(C)** A bar plot illustrating the ratio of cell clusters in the FRS high and FRS low COAD tumors. **(D)** Multiple volcano exhibiting the changes in markers across various cell types. **(E)** A violin plot displaying the expression levels of cytotoxic molecules in T effector cells among specified groups. **(F)** Pseudotime analysis demonstrating the differentiation trajectory of T cell subsets. **(G)** The network diagram illustrating the interactions of markers associated with T cell function. **(H)** KEGG analysis revealing the enriched signaling pathways.

In our investigation of the influence of the FRS on T cell dynamics, we conducted a comprehensive analysis of scRNA-seq data from the GEO database. Using unsupervised clustering techniques, we analyzed T cell populations and identified seven primary clusters. By annotating these clusters with characteristic markers, we delineated six major T cell subsets: naïve T cells, regulatory T cells (Tregs), effector T cells (Teffs), central memory T cells (Tcms), natural killer T cells (NKTs), and exhausted T cells (Tex). Each subset exhibited distinct expression patterns of specific markers (**Figures 4A, D**, **Supplementary Figure S4A, B**). Our results revealed a notable shift in the proportion of T cell subsets between tumor tissues with high FRS scores and those with low FRS scores. Specifically, there was a significant increase in the proportion of Teff and NKT in tumors with low FRS scores, whereas the proportion of Tex was markedly reduced in comparison to tissues with high FRS scores (**Figures 4B, C**). Given the pivotal role of T cell function in determining the success of tumor immunity, we further analyzed the impact of FRS on the functionality of Teff cells. We observed a significant upregulation of effector molecules in Teff cells within tumors with low FRS scores, including PRF1, GZMH, GZMB, and IFNG (**Figure 4E**). Additionally, we utilized pseudotime analysis to elucidate the temporal evolution of T cell subset differentiation. Our findings indicated that in the early stages of differentiation, the T cell population was predominantly composed of Treg cells. As pseudotime progressed, there was a transition towards a predominance of naive T cells. In the late stages of differentiation, the population was characterized by an increase in Tex cells and NKT cells (**Figures 4F**, **Supplementary Figure S4C**). Importantly, the expression levels of CD8, GZMB, and IFNG and other markers associated with T cell function were significantly increased in the late stages of differentiation (**Supplementary Figures S4D, E**).

Utilizing the transcriptomic sequencing data from the TCGA COAD cohort, we embarked on a comparative analysis of gene expression differences between tumor tissues with high and low levels of FRS score. This analysis unveiled a spectrum of differentially expressed genes (DEGs), which we subsequently subjected to a co-expression network analysis and Kyoto Encyclopedia of Genes and Genomes (KEGG) enrichment analysis. Notably, genes such as NRG1, SAMD13, MFAP3L, SALL1, ZNF704, SEMA5A, and HLA-DQB1 were identified as having altered expression patterns, potentially reflecting the diverse roles of FGFRS in tumor biology. The presence of immune-related genes such as CX3CL1, HLA-DQBY, and KIR2DL4, along with cytotoxic markers like GZMH and GZMB, suggests an intricate interplay between FRS signaling and immune response modulation within the TME (**Figure 4G**). Furthermore, the KEGG analysis elucidated the biological pathways and cellular processes significantly enriched among the differentially expressed genes (DEGs). Pathways such as the NF-kappa B signaling pathway, the STAT signaling pathway, and the TNF signaling pathway were prominently highlighted, indicating the roles of immune response and cell signaling in tumors altered by the FRS. Additionally, the identification of processes, including neutrophil extracellular trap formation and natural killer cell-mediated cytotoxicity, underscores the influence of the FRS on immune cell functions (**Figure 4H**). Collectively, FRS exhibited a negative correlation with T cell function, thereby impairing antitumor immunity and facilitating tumor progression.

### FRS correlates to ICB resistance in murine MC38 tumor model

**Figure 5:**
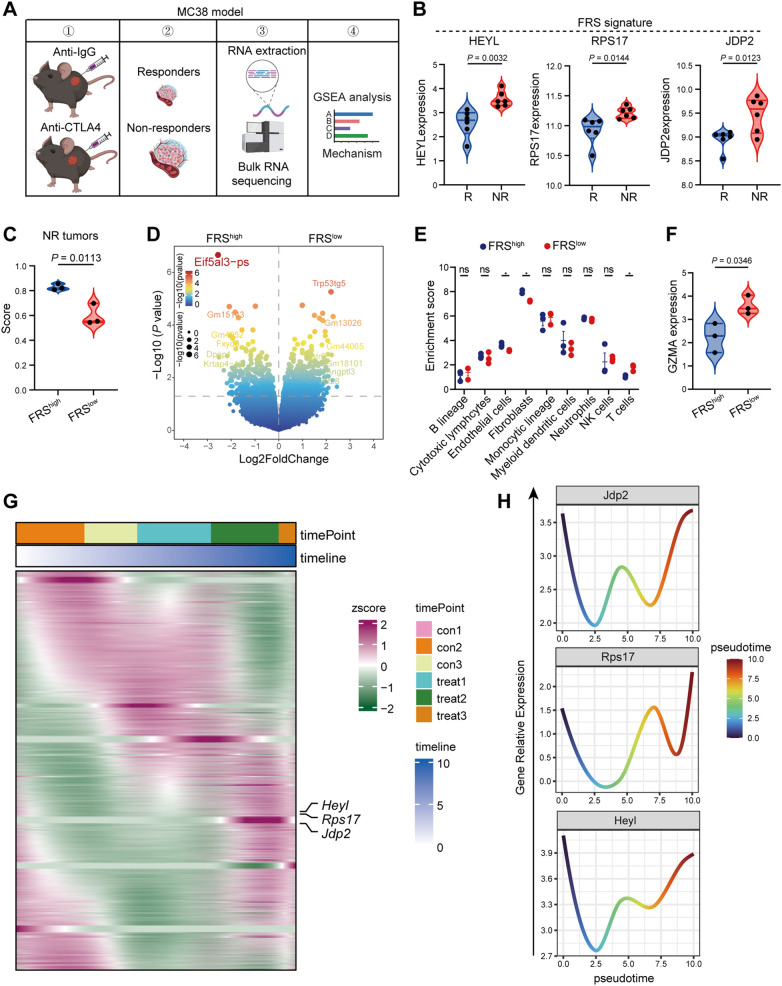
High FRS score correlates positively with ICB resistance in murine MC38 tumors. **(A)** The schematic representation of the experimental timeline for the RNA-seq analysis of ICB resistant murine MC38 tumors. The image was illustrated by the Biorender.com. **(B)** Expression profiles of pivotal genes within the FRS in responsive and nonresponsive MC38 tumors, highlighting variations in gene activity, R, responders, NR, non-responders. **(C)** Violin plot depicting the distribution of scores for tumors with high and low expression of FRS, specifically within the nonresponsive MC38 tumor group. **(D)** A volcano plot illustrating the differential gene expression between tumors exhibiting high versus low FRS score in the nonresponsive category. **(E)** MCPcounter analysis revealing the enrichment of distinct cell clusters associated with high and low FRS score in nonresponsive tumors. **(F)** A violin plot displaying the expression levels of GZMB in tumors with high and low FRS score among the nonresponsive group. **(G)** Heatmap representing the expression patterns of key genes derived from pseudotime analysis of bulk RNA-seq data from nonresponsive tumors. **(H)** Line graphs illustrating the trend of key gene expression changes within the FRS across the nonresponsive tumors. P values are from unpaired t-tests **(B, C, F)** and two-way ANOVA **(E)**.

The expression of FRS is inversely associated with T cell function, but it is unclear whether this correlation contributes to immunotherapy resistance. In order to elucidate the role of FRS in colon tumors following immune checkpoint blockade (ICB) therapy, we developed an ICB-resistant MC38 tumor model and conducted RNA sequencing analysis. Within our experimental framework, mice engrafted with MC38 tumors were treated with either IgG or anti-CTLA4 antibodies, and we meticulously monitored changes in tumor volume. Upon completion of the treatment, we classified tumors that exhibited a significant reduction in volume following anti-CTLA4 treatment as “responder” phenotypes, in stark contrast to those that showed minimal change with IgG treatment, which we labeled as “nonresponder” phenotypes (**Figure 5A**). Of particular interest, the expression levels of HEYL, RPS17, and JDP2 were significantly elevated in tumors from nonresponders compared to those from responders (**Figure 5B**). To investigate the potential correlation between FRS expression and immune checkpoint blockade (ICB) resistance, we stratified nonresponders into two groups based on the gene set variation analysis (GSVA) score of the FRS and identified differentially expressed genes (DEGs) between high and low FRS-expressing tumors (**Figures 5C, D**). Utilizing these DEGs, we performed GSEA, revealing that several signaling pathways were significantly enriched in low FRS -expressing tumors, including those involved in extracellular matrix organization, collagen formation, and oxidative stress response. Notably, key genes associated with collagen and oxidative stress were found to be significantly downregulated in low FRS -expressing tumors (**Supplementary Figures S5A-F**). Further investigation into the impact of FRS on the TME of nonresponsive MC38 tumors using MCPcounter analysis indicated elevated levels of endothelial and fibroblast infiltration, alongside a diminished presence of T cells in the TME of tumors with high FRS expression (**Figure 5E**). Additionally, the expression of GZMA, a marker for cytotoxic T lymphocytes, was significantly higher in tumors with low FRS expression (**Figure 5F**). Our data suggest that reduced FRS may enhance T cell function within the TME. To comprehensively assess the influence of FRS expression on ICB-resistant tumor development, we conducted pseudotime analysis on RNA sequencing data from nonresponsive tumors. The findings revealed that the expression patterns of three key genes closely mirrored one another and were significantly elevated during the advanced stages of tumor progression (**Figures 5G, H**). Collectively, these results suggest that the FRS is positively associated with ICB resistance and may facilitate tumor progression.

### FRS inhibitor significantly rewires the TME to promote tumor regression

**Figure 6:**
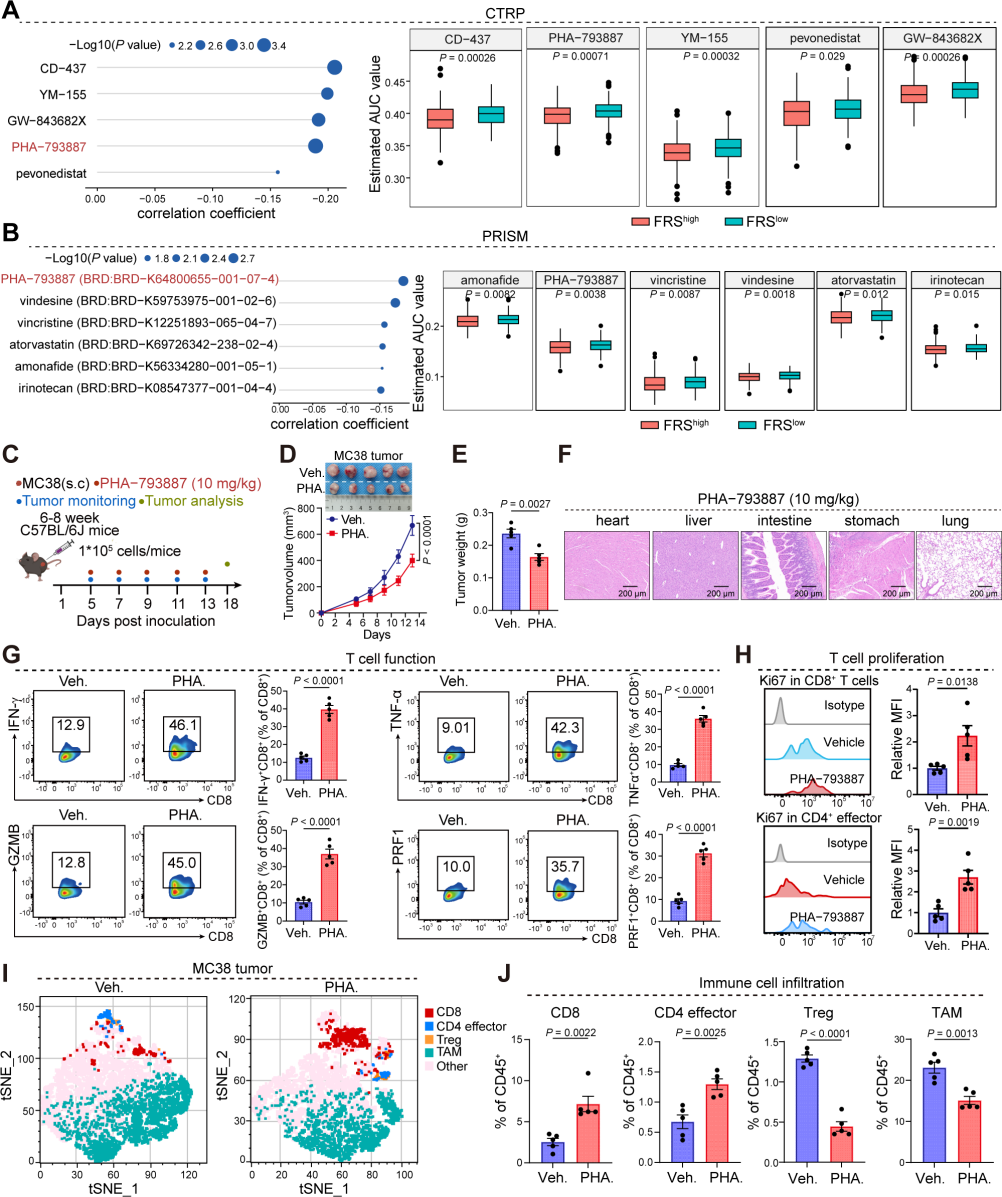
PHA-793887 enhances antitumor immunity in MC38 tumor model. **(A, B)** The prediction of drugs targeting FRS via CTRP **(A)** and PRISM **(B)** database. **(C)** The schedule process of animal experiments. **(D, E)** Tumor growth **(D)** and tumor weight **(E)** of MC38 tumors bearing mice treated with Veh. or PHA. (10 mg/kg). Veh., vehicle; PHA., PHA-793887. **(F)** Representative images of hematoxylin and eosin (HE) staining were obtained to evaluate organ toxicity in mice treated with either vehicle or PHA-793887. **(G)** The expression levels of cytotoxic molecules in the CD8^+^ T cells within MC38 tumors treated with Veh. or PHA. **(H)** The expression of ki67 in the CD8^+^ T cells (top) and CD4 effector cells (bottom). **(I)** tSNE plot showing the distribution of immune cell clusters. Distinct colors represent the different cell clusters. **(J)** The box plot showing the percentages of CD8^+^ T cells, CD4 effector cells, Treg cells, and TAMs in the MC38 tumors treated with Veh. or PHA, Treg, regulator T cell, TAM, tumor-associated macrophage, Veh., Vehicle, PHA., PHA-793887. *P* values are from a two-tailed unpaired Student’s t-test **(A, B, E, G, H, J)** and two-way ANOVA **(D)**.

As our analysis, the correlation between FRS expression and tumor immunity has been established, yet the extent to which FRS inhibitors can curtail tumor progression through the enhancement of antitumor immunity is not fully understood. To address this, we utilized the CTRP and PRISM databases to prognosticate potential inhibitors that target the FRS and subsequently validated the antitumoral efficacy of these inhibitors in murine tumor models. Through the amalgamation of data from both databases, we identified PHA-793887, recognized as a quintessential cyclin-dependent kinase (CDK) inhibitor, as a promising candidate with potential inhibitory effects on the FRS (**Figures 6A, B**). Then, we developed a mouse xenograft model of colorectal cancer by subcutaneously inoculating MC38 tumor cells and evaluated the impact of PHA-793887 on tumor progression and the TME via oral administration (**Figure 6C**). Notably, the treatment did not induce any discernible harm to the vital organs of the mice, encompassing the heart, liver, intestine, stomach, and lungs (**Figure 6D**). Importantly, the PHA-793887 significantly suppressed tumor growth and reduced the tumor burden relative to the control cohort (**Figure 6E**). Further examination of the TME utilizing flow cytometry disclosed that PHA-793887 markedly augmented the secretion of cytotoxic T cell-associated cytokines, including interferon-gamma (IFN-γ), tumor necrosis factor-alpha (TNF-α), and granzyme B (GZMB), within the tumor milieu (**Figure 6F**). This suggests an enhancement of T cell functionality. Moreover, in tumors treated with PHA-793887, there was a significant increase in the infiltration of CD8^+^ T cells and effector CD4^+^ T cells, along with a decrease in tumor-associated macrophages (TAMs) and regulatory T cells (Tregs) (**Figures 6G–J**). These findings underscore that PHA-793887, acting as an FRS inhibitor, can profoundly rewire the immunosuppressive TME, bolster anti-tumor immune responses, and reduce tumor growth.

### FRS is positively correlated ICB therapy resistance in cancer patients

**Figure 7:**
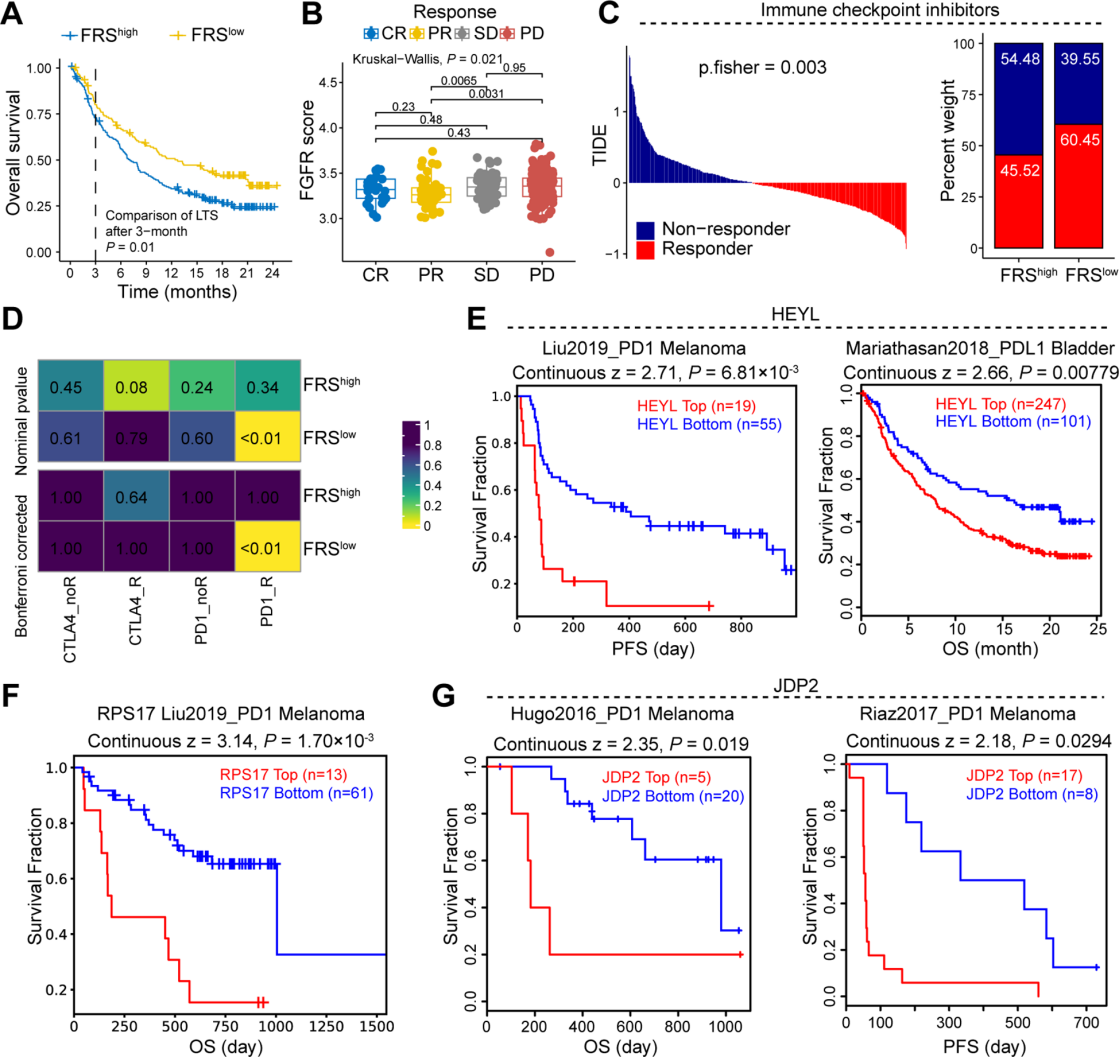
FRS is negatively correlated to the response of immune therapy in cancer patients. **(A)** Survival curve of cancer patients with high or low FRS score. **(B)** The FRS score of cancer patients in the indicated groups, CR, complete response, PR, partial response, SD, stable disease, PD, progressive disease. **(C)** TIDE analysis showing the immune therapy response of cancer patients. **(D)** Submap showing the correlation between FRS score and therapy response of ICB in the cancer patients. **(E-G)** The survival analysis of cancer patients received ICB therapy in indicated groups, PFS, progression-free survival, OS, overall survival. P values are from the log-rank test **(A, E-G)** and one-way ANOVA **(B)**. Pearson’s correlation coefficient is calculated **(D)**.

To further elucidate the association between FRS and the therapeutic efficacy of ICB in cancer patients, we conducted an integrative analysis using TCGA and the Tumor Immune Dysfunction and Exclusion (TIDE) database. Our analysis revealed that patients with tumors exhibiting high FRS score had a significantly lower survival rate compared to those with low FRS score within their tumors (**Figure 7A**). Additionally, by examining patients within the TIDE database who underwent ICB treatment, we observed a notable increase FRS among patients with stable disease (SD) or progressive disease (PD) compared to those with partial response (PR) (**Figure 7B**). Utilizing the TIDE scoring system, we identified a higher prevalence of responders among patients with low FRS expression (**Figure 7C**). Furthermore, the SubMap analysis revealed that the low FRS group exhibited a high likelihood of response to anti-PD-1 in the immunotherapy cohorts (IMvigor210) (**Figure 7D**). In the context of patients undergoing ICB therapy, we conducted a detailed analysis of the prognostic impact of key genes within the FRS. Our findings indicate that tumors with elevated expression levels of HEYL, RPS17, and JDP2 are associated with a poorer prognosis for cancer patients (**Figures 7E-G**). These results suggest that the FRS may serve as a prognostic indicator for ICB treatment outcomes, with a lower FRS score potentially correlating with a more favorable response to ICB therapy.

## Discussion

Our comprehensive analysis leveraging scRNA-seq data from CRC cohorts has unveiled the intricate relationship between FRS and tumor biology, particularly within the TME. The identification of FGFRS^+^ fibroblast subsets through scRNA-seq analysis provides a foundation for understanding the heterogeneity of cancer and the potential for targeted therapeutic intervention. The significant association between FRS and fibroblasts suggests a pivotal role for these cells in modulating the TME, which could be a critical factor in tumor progression and response to therapy. The enrichment of FRS in fibroblasts, as evidenced by the highest module scores, indicates that these cells may be key players in the tumor’s resistance to ICB therapy. This finding is supported by the observation that FGFRS^+^ fibroblasts display distinct gene expression profiles linked to pathways, including epithelial tube morphogenesis and collagen fibril organization, which are known to influence tumor growth and invasion.

Previous studies have reported the relevant signature of immune cells, such as macrophages, predicted patient prognosis and therapy resistance (16–19). The development of a predictive model using machine learning algorithms has been instrumental in identifying a gene signature that strongly predicts patient survival outcomes (20, 21). The FRS, composed of genes like JDP2, HEYL, NRG1, RPS17, and MANF, has demonstrated robust predictive capabilities across various cohorts. This underscores the potential of using FRS as a prognostic tool in clinical settings to stratify patients into high and low-risk groups, thereby personalizing treatment strategies. Furthermore, our analysis elucidates the correlation between the FRS and tumor immunity. The identification of distinct patient clusters based on FRS, along with the associated differences in immune scores and cell infiltration patterns, suggests a complex interplay between the FRS and immune cell dynamics within the TME. Notably, the reduced infiltration of CD8^+^ T cells and the increased presence of M0-like macrophages in tumors with higher FRS scores indicate a possible mechanism through which FRS enrichment may suppress antitumor immunity.

Many drugs harbor significant anti-tumor, whereas the mechanism remains elusive (22). Previous research has established PHA-793887 as a potent, ATP-competitive cyclin-dependent kinase (CDK) inhibitor capable of inhibiting key cell cycle regulators such as CDK2, CDK1, CDK4, AND CDK9 (23, 24). While its efficacy in disrupting cell cycle progression is well-documented, the exploration of additional potential targets and its impact on tumor immunity remains less explored (25). Our study takes a significant step toward addressing this gap by employing in silico drug prediction methods to identify FRS as a potential target for PHA-793887. The application of PHA-793887 in our murine model of colorectal cancer has yielded promising results, demonstrating a significant inhibitory effect on tumor growth without any detectable organ toxicity. In the TME, various factors could contribute to T cell dysfunction, subsequently promoting immunotherapy resistance and tumor progression (26, 27). In this study, we found PHA-793887 treatment induced an enhanced secretion of cytotoxic T cell-associated cytokines and facilitated an increase in the infiltration of CD8^+^ T cells and effector CD4^+^ T cells within the TME. These observations suggest an improvement in T cell functionality, indicative of the compound’s potential to bolster antitumor immunity. Together, these findings underscore the PHA-793887 could be a potential inhibitor enhancing antitumor immunotherapy for the cancer patients with CRC. Future research should focus on clinical trials to evaluate the efficacy of FRS inhibitors in combination with ICB therapies and on further elucidating the mechanisms by which FRS modulates the TME and immune responses in CRC patients.

## Conclusion

Our study presents a multi-faceted perspective on the role of FRS in colorectal cancer, highlighting its enrichment in fibroblasts, its correlation with ICB resistance, and its impact on tumor immunity. The findings have implications for the development of novel therapeutic strategies targeting the FRS and for the refinement of prognostic models to better predict patient outcomes. Importantly, PHA-793887 could be a potential inhibitor targeting the FRS in the immunotherapy of CRC patients.

## Data Availability

The original contributions presented in the study are included in the article/**Supplementary Material**. Further inquiries can be directed to the corresponding author. The public databases presented in the study are deposited in the Gene Expression Omnibus repository (http://www.ncbi.nlm.nih.gov/geo/), accession number: GSE231559, GSE166555, GSE17536, GSE29621, and GSE38832.
