# Supplementary material for: Fibroblast growth factor receptor risk signature predicts patient prognosis and immunotherapy resistance in colorectal cancer
Source: Front Immunol. 2024 Nov 29;15:1493673. doi: 10.3389/fimmu.2024.1493673 (PMC11638221; doi:10.3389/fimmu.2024.1493673)
Supplement: Supplementary file 1 [file DataSheet1.docx]

**Supplementary Figures for Fibroblast growth factor receptor risk signature predicts patient prognosis and immunotherapy resistance in colorectal cancer**

**Xiaofang Li^1#^, Zhiling Pan^2,3#^, Tiankuo Luan^4#^, Qian Xiao^4^, Liuying Li^4^, Qianxue Wu^4^, Guoqing Yao^5^, Xiang Zhang^6*^ and Daqiang Song^4*^**

**Supplementary Figure 1-5**

**
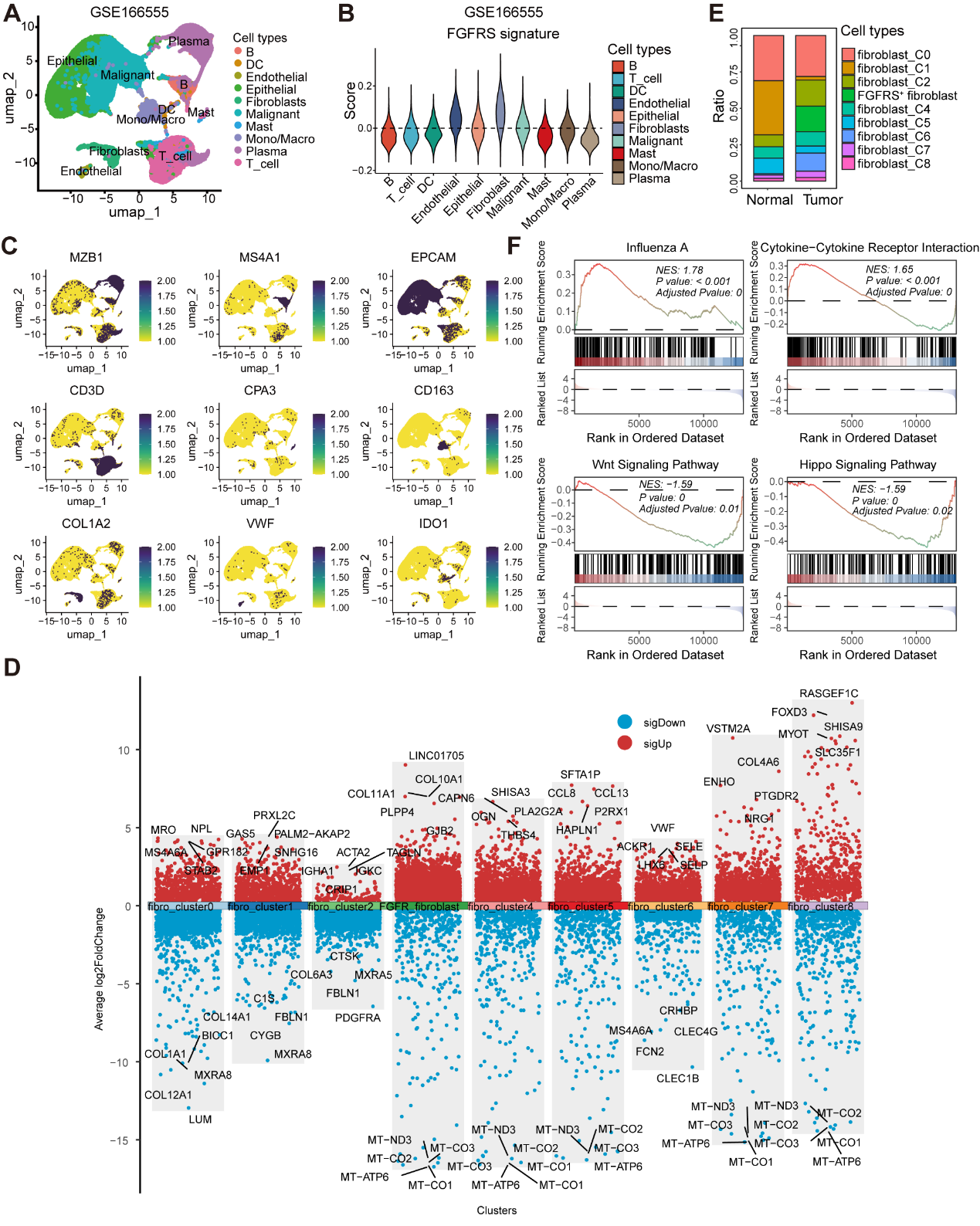
**

**Figure S1. The scRNA-seq analysis reveals the FGFRS-positive cell clusters.**

(A) UMAP showing the cell clusters in the CRC cohort from GEO database. Distinct colors represent the different cell clusters. (B) Violin plot showing the FGFRS score in the indicated cell clusters. (C) UMAP showing the expression of representative markers of distinct cell types. (D) Multiple volcano plot showing the expression of differential genes in the distinct cell clusters. (E) The ratio of fibroblast subsets. (F) GSEA analysis showing the enriched signaling pathways in the FGFRS positive fibroblasts. *P* values are from a two-tailed unpaired Student’s t-test (D, F).


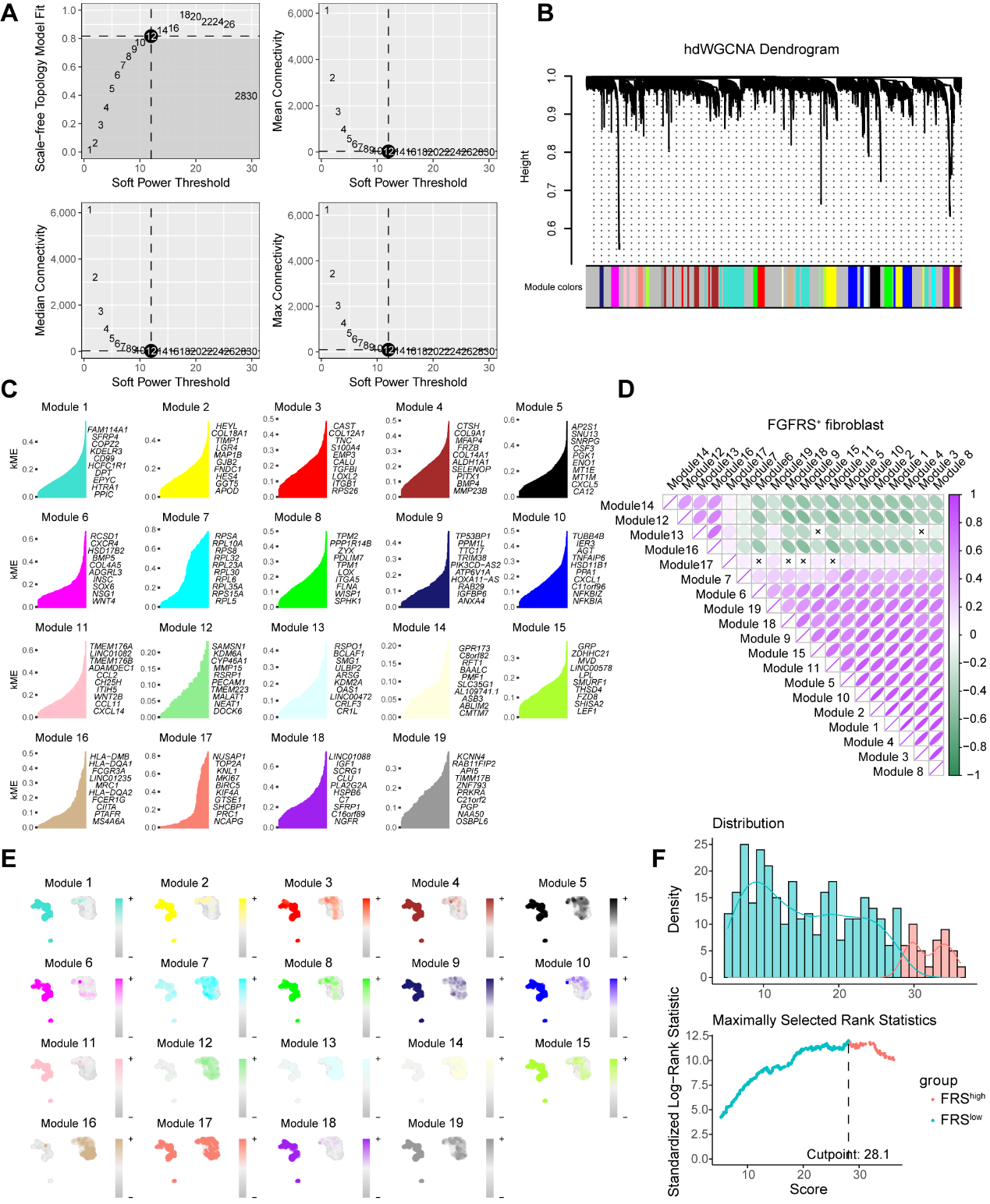


**Figure S2. hdWGCNA analysis identifies the key molecule modules correlated FGFRS fibroblasts.**

(A) The selection of soft-thresholding powers and the impact of these powers on the scale-free topology fit index and average network connectivity across different weighting coefficients are discussed. (B) The dendrogram illustrating the hierarchical clustering of gene modules in hdWGCNA, with module colors indicating distinct groups of co-expressed genes. The height of the dendrogram branches represents the dissimilarity between modules. (C) Three FGFR-fibroblast-related gene modules were identified, and the top hub gene is presented according to the hdWGCNA pipeline. (D) The matrix plot visually represents the inter-module relationships by depicting the correlation between module eigengenes. (E) UMAP plots as in Figure 1D, colored by MEs for the 19 co-expression gene modules. (F) The score distribution from a RSF analysis, with maximally selected rank statistics indicating the strength of variable importance.


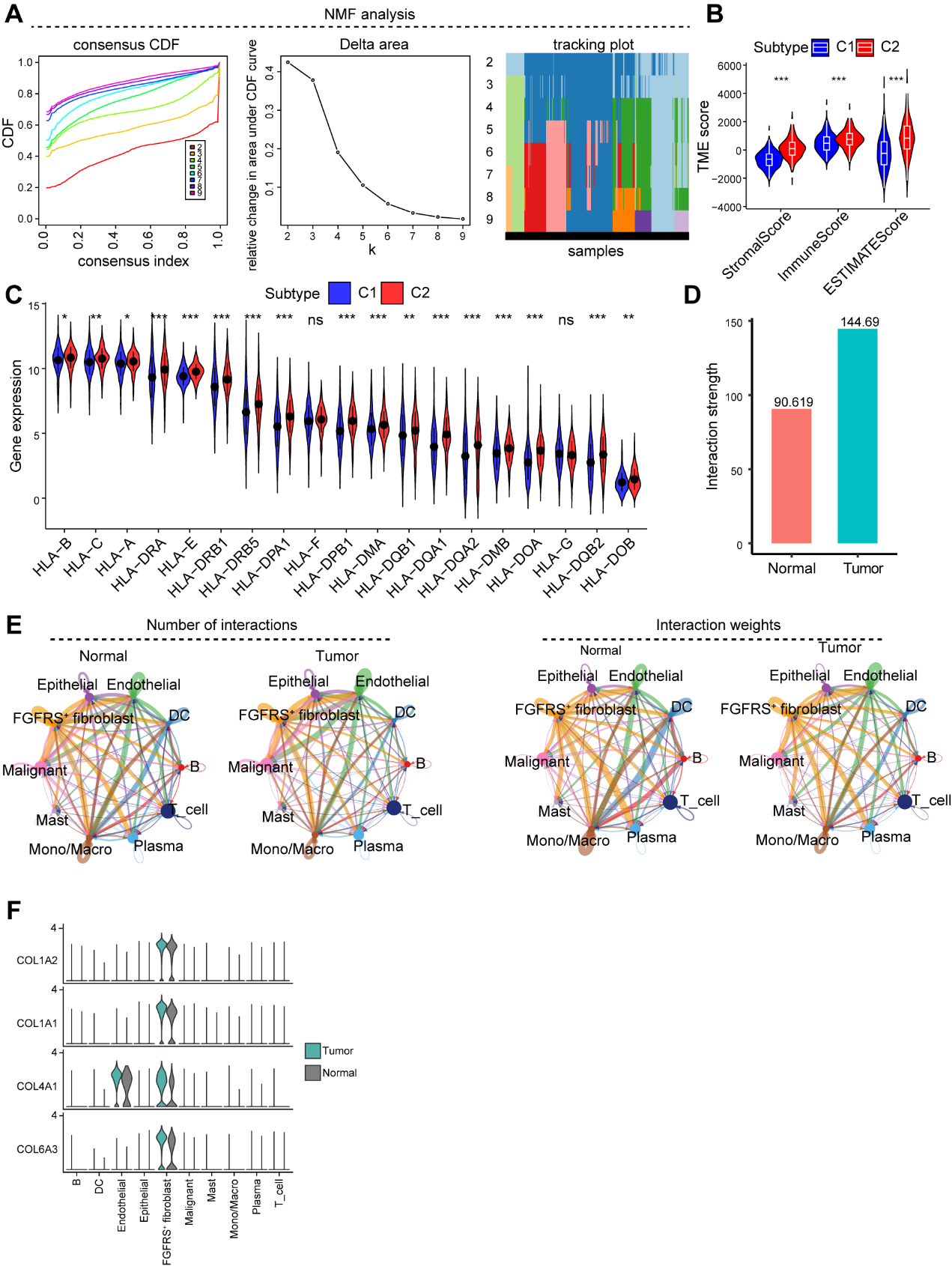


**Figure S3. FGFRS molecule subtypes is associated with tumor immunity.**

(A) The NMF analysis plot shows the tracking of consensus indices, with the relative change in the area under the CDF curve indicating subtype stability, NMF, non-negative matrix factorization, CDF, cumulative distribution function. (B) Violin plot showing the TME score, C1, cluster 1, C2, cluster 2. (C) Violin plot showing the expression levels of immune activation relevant genes in the FGFRS subtype. (D) The interaction strength in the normal or COAD tumors. (E) The network showing the number and weight of interaction of cells in the COAD tumors. (F) Violin plot showing the expression of collagen related genes in the normal and tumor tissues.


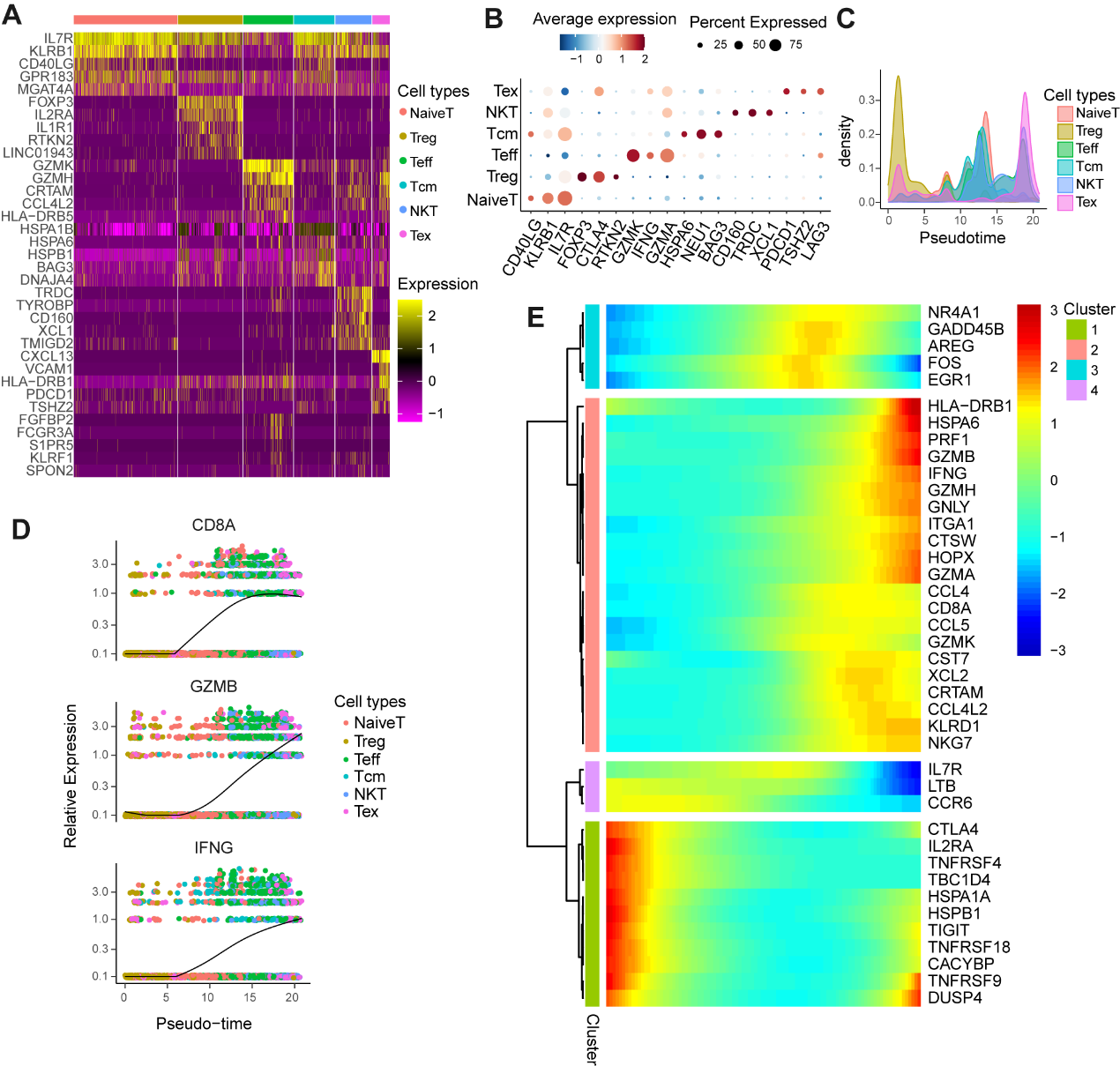


**Figure S4. The scRNA-seq analysis shows the marker expressions of T cell subset.**

(A) The heatmap showing the expression of top 5 markers in the distinct cell clusters, Treg, regulator T cells, Teff, effector T cells, Tcm, central memory T cells, NKT, natural killer cells, Tex, exhausted T cells. (B) Dot plot showing the established markers in the cell types. (C) The density of cell clusters in the pseudotime. (D) The expression of selected genes in different cell states as the pseudotime progresses. (E) Heatmap showing the dynamic changes in gene expression of the different cell clusters.


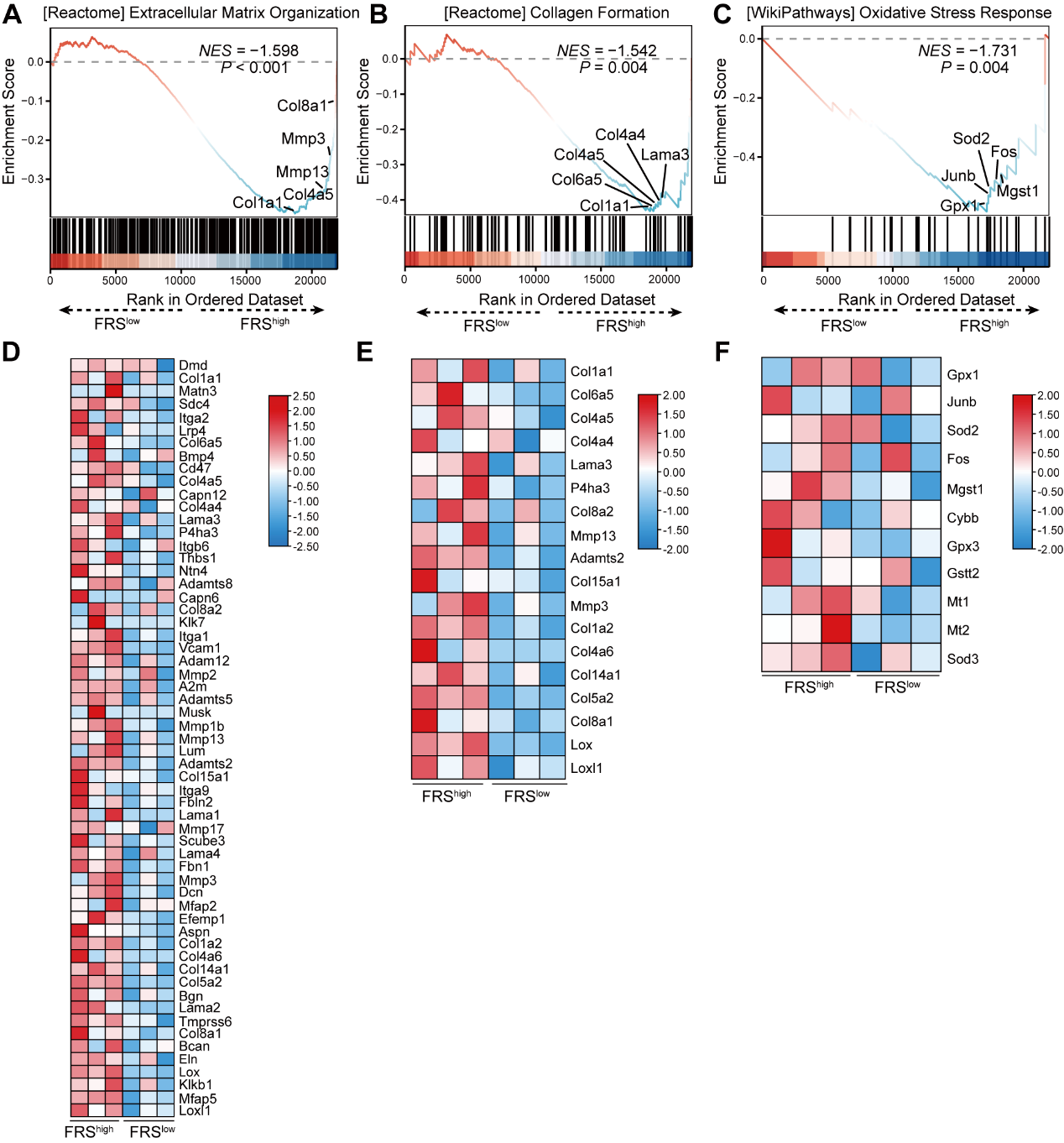


**Figure S5. GSEA analysis reveals enriched signaling pathways in the ICB resistant MC38 tumors.**

(A-C) Signaling pathways enriched in the high and low FRS nonresponsive MC38 tumors, NES, normalized enrichment score. (D-F) Heatmap showing the key gene expression in the enriched signaling pathways.
